# Tracking by segmentation with future motion estimation applied to person-following robots

**DOI:** 10.3389/fnbot.2023.1255085

**Published:** 2023-08-28

**Authors:** Shenlu Jiang, Runze Cui, Runze Wei, Zhiyang Fu, Zhonghua Hong, Guofu Feng

**Affiliations:** ^1^School of Computer Science and Engineering, Macau University of Science and Technology, Macao, Macao SAR, China; ^2^College of Information Technology, Shanghai Ocean University, Shanghai, China; ^3^College of Surveying and Geo-Informatics, Tongji University, Shanghai, China

**Keywords:** person-following robot, tracking by segmentation, mobile robot, visual tracking, deep learning

## Abstract

Person-following is a crucial capability for service robots, and the employment of vision technology is a leading trend in building environmental understanding. While most existing methodologies rely on a tracking-by-detection strategy, which necessitates extensive datasets for training and yet remains susceptible to environmental noise, we propose a novel approach: real-time tracking-by-segmentation with a future motion estimation framework. This framework facilitates pixel-level tracking of a target individual and predicts their future motion. Our strategy leverages a single-shot segmentation tracking neural network for precise foreground segmentation to track the target, overcoming the limitations of using a rectangular region of interest (ROI). Here we clarify that, while the ROI provides a broad context, the segmentation within this bounding box offers a detailed and more accurate position of the human subject. To further improve our approach, a classification-lock pre-trained layer is utilized to form a constraint that curbs feature outliers originating from the person being tracked. A discriminative correlation filter estimates the potential target region in the scene to prevent foreground misrecognition, while a motion estimation neural network anticipates the target's future motion for use in the control module. We validated our proposed methodology using the VOT, LaSot, YouTube-VOS, and Davis tracking datasets, demonstrating its effectiveness. Notably, our framework supports long-term person-following tasks in indoor environments, showing promise for practical implementation in service robots.

## 1. Introduction

Person-following robots have emerged as a crucial application in various domains due to their capacity for accurate and prompt target tracking. Existing methodologies predominantly use classifiers that are constructed using the tracking-by-detection strategy, often yielding a basic region of interest (ROI) that fails to comprehensively represent the complex human form (Yoshimi, 2006; Cosgun et al., 2013; Cheng et al., 2019).

To augment the precision and responsiveness of these robotic applications, the present research introduces a novel end-to-end Deep Neural Network (DNN) for person-following. This approach enables pixel-level tracking of the target individual and integrates a real-time future motion estimation function, facilitating the robot's capacity to anticipate and swiftly react to the individual's movements (Lin et al., 2012; Cosgun et al., 2013; Cheng et al., 2019; Koide et al., 2020; Hu et al., 2022).

The proposed DNN is characterized by three main components: (1) Person tracking via segmentation for real-time tracking; (2) A classification-lock pre-trained feature layer to mitigate the influence of environmental noise; and (3) A future motion estimation module for rapid prediction of the individual's movements, thereby informing the control module.

The “tracking-by-segmentation” strategy incorporated in our tracker allows for pixel-level tracking of the target, providing a significantly more detailed target representation compared to traditional ROI output methods. The classification-lock pre-trained layer contributes to the recognition of discriminative models, thereby strengthening the system's tracking accuracy, particularly in scenarios where the human appearance undergoes substantial transformations (Held et al., 2016; Caelles et al., 2017; Gundogdu and Alatan, 2018; Paral et al., 2019).

The tracker incorporates discriminative correlation filters and a CL-pre-trained layer to provide resilience to common tracking challenges, such as occlusion and dramatic changes in appearance. Furthermore, the inclusion of a future motion estimation neural network bestows the robot with the capability to anticipate the target's motion pattern, ensuring quicker and more effective response times compared to standard trackers.

We conducted a comprehensive evaluation of our proposed method through three major experiments. These include testing our tracker on the VOT (Kristan et al., 2018), VOS (Xu et al., 2018), and DAVIS (Perazzi et al., 2016) datasets, real-world robot operations, and a practical following task in an indoor environment. In all of these tests, our method demonstrated superior performance, effectively detecting the speed and direction of the target and minimizing rotation errors during person-following.

The primary contributions of this research are as follows:

1. The “tracking-by-segmentation” approach significantly enhances the accuracy and robustness of the tracking system.
2. The classification-lock pre-trained layer improves the system's ability to recognize discriminative models.
3. The incorporation of discriminative correlation filters and a CL-pre-trained layer provides the tracker with resilience to challenges such as occlusion and dramatic changes in appearance.
4. The future motion estimation module facilitates quick and effective responses.
5. Our approach operates independently of multiple human tracking technologies.
6. The robustness and accuracy of our method even enable the operation of a robot with a basic control method such as a PID controller within a complex indoor environment.

To emphasize the practical implications and the unique advantages of our approach, we have included a demonstration of the operation of our method at the end of the paper. The value of our work lies not only in the application of deep learning to tracking, but also in the specific innovations and strategies introduced. Through the strategic blend of classification-lock tracking, future motion estimation, and the use of deep learning techniques, we offer a novel solution to address the challenges of robot person-following, thereby improving the accuracy and robustness of the system.

## 2. Related works

The pursuit of robust and efficient person-following robots necessitates a comprehensive understanding and leveraging of visual tracking, object segmentation, and motion planning strategies. This study introduces an innovative methodological approach to improving the effectiveness of person-following robots in terms of both accuracy and responsiveness. The proposed solution is predicated on several critical components: a novel visual tracker, a video object segmentation and tracking strategy, a pixel-level classification-lock mechanism, the implementation of discriminative correlation filters (DCF), and a future motion estimation mechanism.

Traditionally, person-following robots have used region of interest (ROI) to represent a person's position within a scene. However, this representation can be unstable due to the different poses a person may assume, leading to significant ROI fluctuations and inaccuracies. Although Deep Neural Networks (DNNs) have been implemented in visual trackers such as Caelles et al. (2017), Wang et al. (2019), Lukezic et al. (2020), and Koide et al. (2020) that produce high frames per second (FPS) ROI trackers, these systems may not adequately track the target during substantial pose variations. There have been efforts to create a more robust representation of a person's position in 3D space using plane and height estimation techniques (Chou and Nakajima, 2017; Jiang et al., 2018; Zou and Lan, 2019; Hu et al., 2022). Still, they predominantly yield an approximation of the person's position.

In the field of object segmentation and tracking within videos, video object segmentation (VOS) has been harnessed for delineating moving objects (Koide and Miura, 2016; Caelles et al., 2017; Voigtlaender and Leibe, 2017; Yang et al., 2018; Voigtlaender et al., 2019; Lukezic et al., 2020; Wu et al., 2021). Nonetheless, these methods often rely on computationally-intensive and time-consuming deep neural networks, posing challenges for mobile robots. Typically, the first frame is used as a reference target, with subsequent frames matched to this initial target. This strategy is susceptible to issues when tracking fast-moving objects or those that undergo significant appearance changes. Some researchers have proposed a two-stage tracking-by-segmentation framework, but such a method is heavily reliant on the ROI generation results and can struggle to adapt to dynamic scenes. To mitigate these shortcomings, we propose a one-stage architecture that directly outputs the clustered mask, which has proven effective in preliminary tests.

A significant challenge for person-following robots is target selection, particularly when the appearance of the target individual changes. To address this, we have introduced a pixel-level classification-lock strategy, forcing the tracker to generate the human appearance as a discriminative model. We propose to lock the human shape as a discriminative target within the tracker using a classification-lock pre-trained feature map. The map utilizes SegNet to initiate the neural network and assists the tracker in constructing a discriminative model of the target individual (Szegedy et al., 2017; Gao et al., 2018; Li et al., 2018; Wang et al., 2018; Howard et al., 2019; Zhang et al., 2021).

In scenarios where there are multiple instances within the same scene, it becomes difficult for the person-following robot to distinguish the foreground from the background. To resolve this issue, we used the well-established architecture of discriminative correlation filters to construct a probability map of the target's anticipated position in the scene in the following frame. This approach enabled the proposed tracker to focus on a distinct target among similar foreground noises, effectively handling overlapping situations during tracking (Wang et al., 2019; Koide et al., 2020; Zhan et al., 2020).

Future motion planning is another crucial aspect for person-following robots to pre-emptively plan their navigation path. Most existing robots, however, react to a person based on position information from the previous few frames. This strategy entails significant computational costs and inefficiencies due to the unpredictability of human motion. We proposed that human motion within a person-following robot scene can be predicted based on the pose, even from a single frame. To achieve this, we fed the output mask of the tracker into a future motion estimation neural network, which reused the pre-trained base net to acquire the target's features. This network then encoded the object into binary features to output the direction and pose information of the target (Zhang et al., 2021).

Our proposed method was subjected to rigorous evaluation through three distinct experiments: performance assessment on the VOT and DAVIS datasets, real-world robot operations, and a real-time tracking task in an indoor environment. In all of these scenarios, our approach exhibited superior performance compared to other state-of-the-art methods. It demonstrated its robustness in detecting the target's speed and direction and reducing rotation errors during person-following tasks. Furthermore, it displayed resilience in overcoming common challenges such as overlap, changes in appearance, lighting conditions, and scale variations. The proposed method, therefore, facilitates long-term person-following in complex environments at high frame rates on mobile platforms, thus eliminating the need for multiple human tracking technologies.

## 3. The proposed tracker

**Figure 1:**
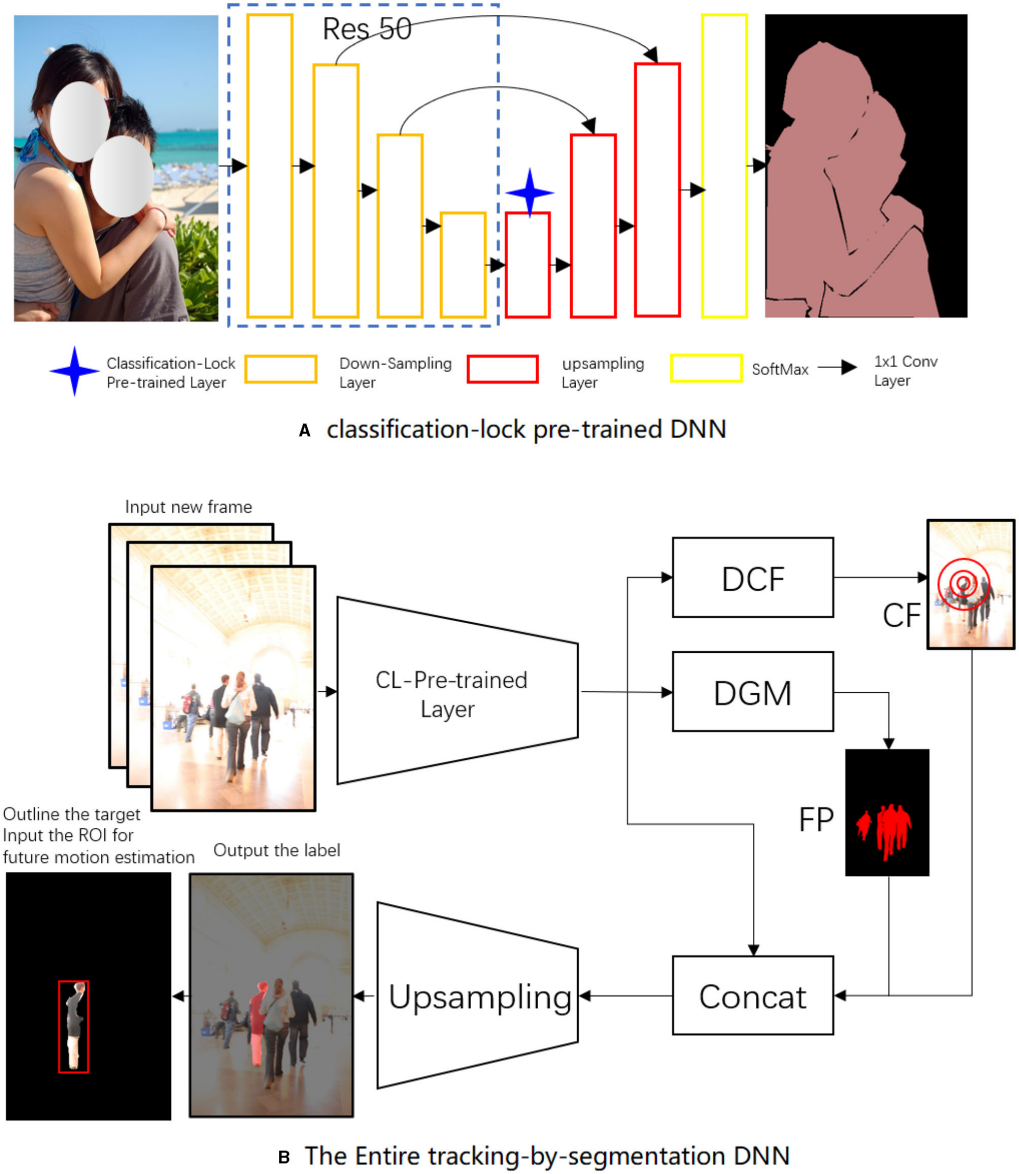
The architecture of the proposed tracker. The discriminative geometric model (DGM) in Figure 2 outputs the foreground posterior (FP). Figure 3 shows the discriminative correlation filter (DCF), which produces a clustered foreground (CF). Figure 4 depicts the upsampling mechanism.

This section provides a comprehensive overview of the proposed tracking system. The general framework of the tracker, as shown in Figure 1, comprises four integral components: a single-shot segmentation tracker, a discriminative correlation filter DNN, a feature merging and upsampling mechanism, and a future motion DNN.

In our method, the foundation was laid by the Single-Shot Segmentation Tracking Neural Network (SSTNN). The SSTNN operates on a “tracking by segmentation” approach, which allows the network to track targets at the pixel level. This strategy provides a fine-grained description of the target, a significant leap from conventional region of interest (ROI) output methods. SSTNN, with its fully convolutional architecture, processes the images in one pass and generates pixel-wise segmentation maps. The precisely delineated boundaries around the target, facilitated by this segmentation, provide the tracking mechanism with increased accuracy.

The classification-lock (CL) pre-trained layer was introduced to ensure the model's resilience to drastic changes in the target's appearance. This layer was meticulously trained to identify human shapes and forms, acting as an insurance policy against the potential anomalies introduced by dramatic changes in the target's appearance. By constraining the model of the target to the human form, the CL pre-trained layer safeguards the tracking process from being derailed due to significant appearance deviations across frames.

The discriminative correlation filter (DCF) acts as the workhorse of our tracking mechanism. DCFs are trained to differentiate between the target and its surroundings. They collaborate with the SSTNN, refining the tracking focus to prevent the mistracking of similar targets and efficiently manage occlusion scenarios. DCFs contribute to improving foreground-background differentiation, thereby increasing tracking accuracy.

These elements, when working in concert, result in robust tracking performance. The SSTNN creates a precise and accurate representation of the target through segmentation. The CL pre-trained layer ensures the consistency of this representation across frames, even in scenarios with substantial appearance changes. Simultaneously, the DCF separates the target from its surroundings, intensifying the tracking focus. Together, they form an adaptive, accurate, and robust tracking system capable of overcoming common challenges in person-following tasks.

### 3.1. Single shot segmentation tracking

Achieving robust target segmentation in a visual tracking DNN necessitates spatial constraints via feature-based discriminative classification. The single shot segmentation tracking neural network introduces a discriminative geometric model (DGM) that employs two feature sets to generate feature vectors related to the target person (foreground) and the background.

The ResNet 50, pre-trained with ImageNet, serves as the backbone neural network, providing robust features for segmentation. As our proposed method is designed for person-following tasks, we fine-tuned the pre-trained basenet with a semantic segmentation DNN, which is pre-trained on a human appearance dataset. We aimed to lock the classification of the target as a person by utilizing a classification-lock pre-trained layer. The dimensional space was decreased using 3 × 3 × 128 and 3 × 3 × 64 convolutional layers (with batch normalization and Relu as defaults).

**Figure 2:**
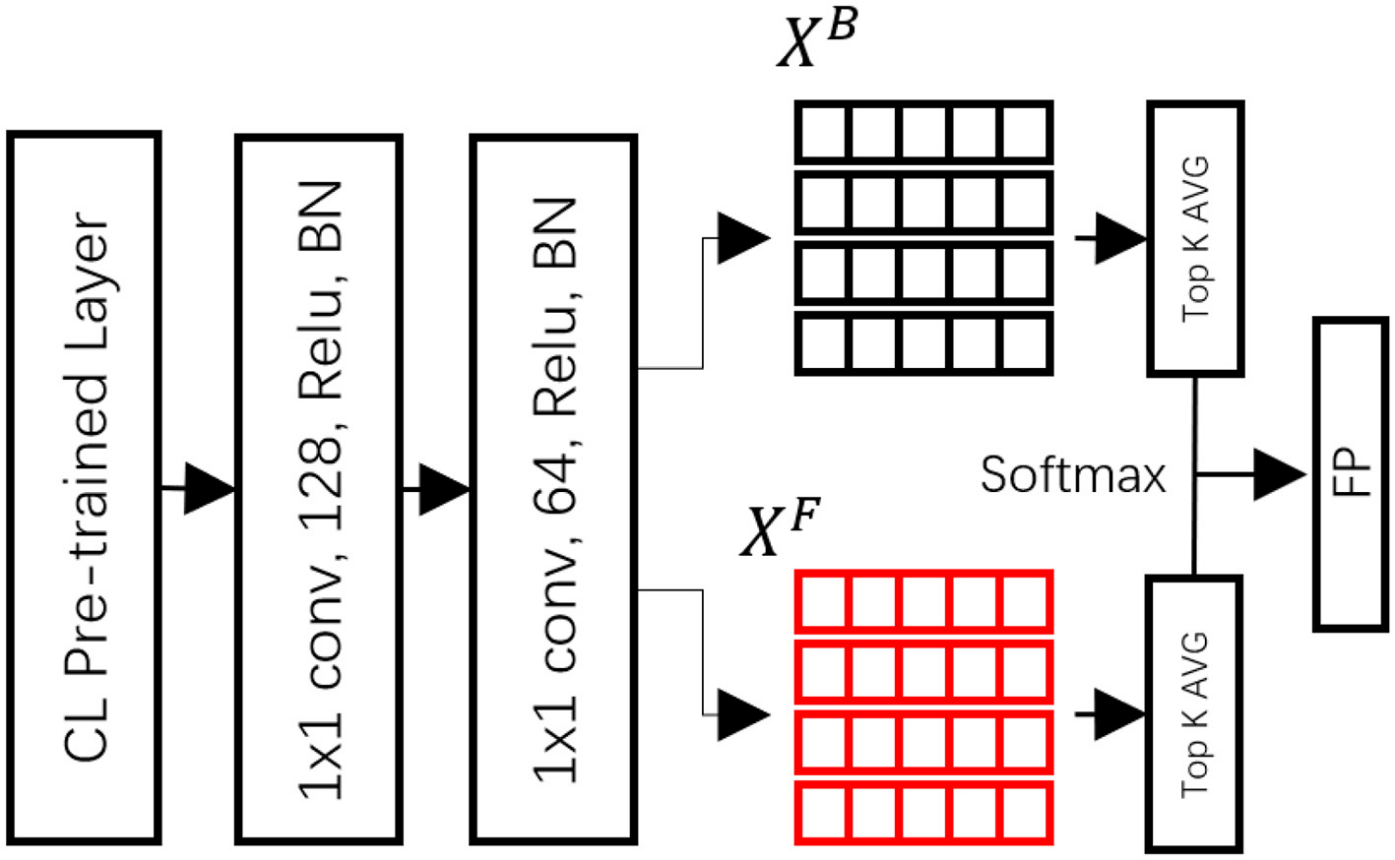
Architecture of the discriminative geometric (DGM) model.

As depicted in Figure 2, the foreground and background models were initialized in the first frame by extracting the foreground feature vectors from the target regions (*X*^*F*^*)* and their surrounding neighborhoods for the background (*X*^*B*^). A region searching algorithm was employed during tracking to compare the extracted foreground and background features with the *X*^*F*^ and *X*^*B*^ of the DGM, utilizing cosine similarity to determine the similarity channels *F* and *B* for tracking.

During tracking, the region searching algorithm was used to compare the extracted foreground and background features to the *X*^*F*^ and *X*^*B*^ of the DGM with cosine similarity to determine the similarity channels F and B for the following. The formula is defined as follows:


(1) $$\begin{array}{c} \;S_{ij}^{F}\left(y_{i},\;x_{j}^{F}\right)=\langle {\tilde{y}}_{i},{\tilde{x}}_{j}^{F}\rangle \end{array}$$

where each feature *y*_*i*_ extracted at pixel *i* is compared to all feature pixels $x_{j}^{F}\in X^{F}$ in the similarity $S_{ij}^{F}$ channel. Here, $\tilde{\left(•\right)}$ represents the *L*_2_ normalization. The final foreground similarity at each pixel *F*_*i*_ is obtained by averaging the top-K similarities at that pixel.


(2) $$\begin{array}{c} F_{i}=TOP\left({\left(S_{ij}^{F}\right\}}_{j=1:N_{F}},K\right) \end{array}$$

where TOP is a top-K averaging operator for a set of *N*_*F*_ similarities. The background similarity channel *B*_*i*_ uses the same formula based on the feature vectors $x_{j}^{B}\in X^{B}$. Through this architecture, the framework leverages features from previous frames to maintain target tracking. With the knowledge from previous frames, the proposed method can manage part/whole occlusion scenarios during tracking, utilizing information from neighboring frames to output the foreground posterior (FP).

### 3.2. Discriminative correlation filter DNN and classification-lock strategy

While the discriminative geometric model (DGM) achieves target-background differentiation via similarity comparison, it can fail under three circumstances: (1) when there is an occlusion in the scenes; (2) when the appearance of objects undergoes significant transformation; and (3) when similar instances are present in the scene. This is largely due to DGM's inability to distinguish between similar instances and its sole reliance on online learning, which is devoid of classification constraints.

To navigate these obstacles, we have incorporated a classification-lock tracking module. The classification-lock (CL) strategy is a core element of our proposed methodology, designed to counter the common problem of losing track during significant changes in the target's appearance. This strategy employs a CL-pre-trained layer that is tailored to the semantic segmentation of the human figure, maintaining the tracking focus regardless of dynamic alterations in the environment. The CL strategy helps to ensure consistent, accurate tracking in real-world scenarios by preventing the system from deviating due to drastic appearance transformations. This module constructs a geometric model that is constrained to a human shape, employing a CL-pre-trained layer and discriminative correlation filters. This allows for an effective adaptation of the target's discriminative features, ensuring accurate tracking despite changes in appearance.

**Figure 3:**
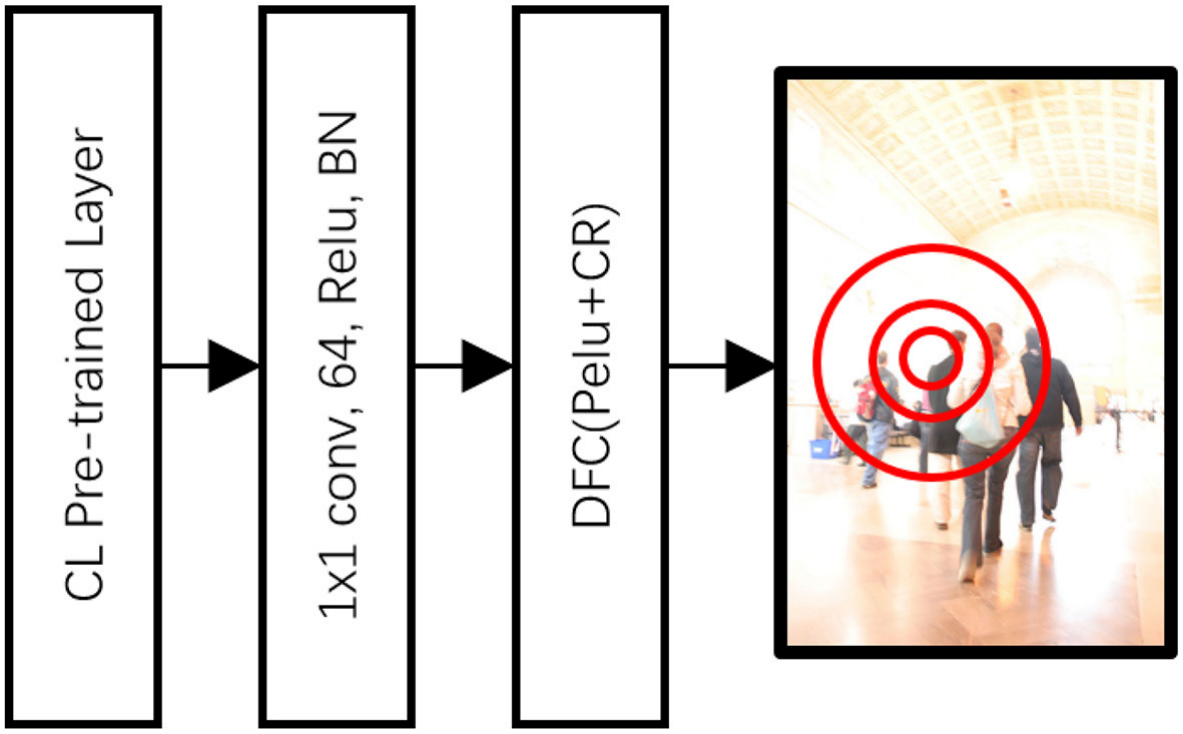
Architecture of the discriminative correlation filter (DCF) DNN.

As seen in Figure 3, the architecture of the Discriminative Correlation Filter (DCF) DNN was configured to ensure that the target's shape was representative of a human form. We utilized the CL pre-trained layer to fine-tune the shape within the semantic segmentation of a person's appearance. This layer is connected to the module through a 1 × 1 × 128 convolutional layer. The DCF module was then utilized, equipped with PeLU nonlinearity and a correlation response, as Kart et al. (2019). The position with the maximum correlation was deemed to be the most similar to the target position. However, given that this method operates on pixel-level features, a probability map generated by a Euclidean distance transform was employed to establish the probable region of the clustered foreground (CF) target.

Through this architecture, the DGM provided discriminative segmentation features based on a few previous frames to segment the discriminative target in the foreground. Meanwhile, the DCF provided the focus of the tracking to avoid mistracking similar targets and manage occlusion scenarios. The CL-pre-trained layer restricted the discriminative target against appearance changes, ensuring accurate and consistent tracking.

### 3.3. Feature merging and upsampling

**Figure 4:**
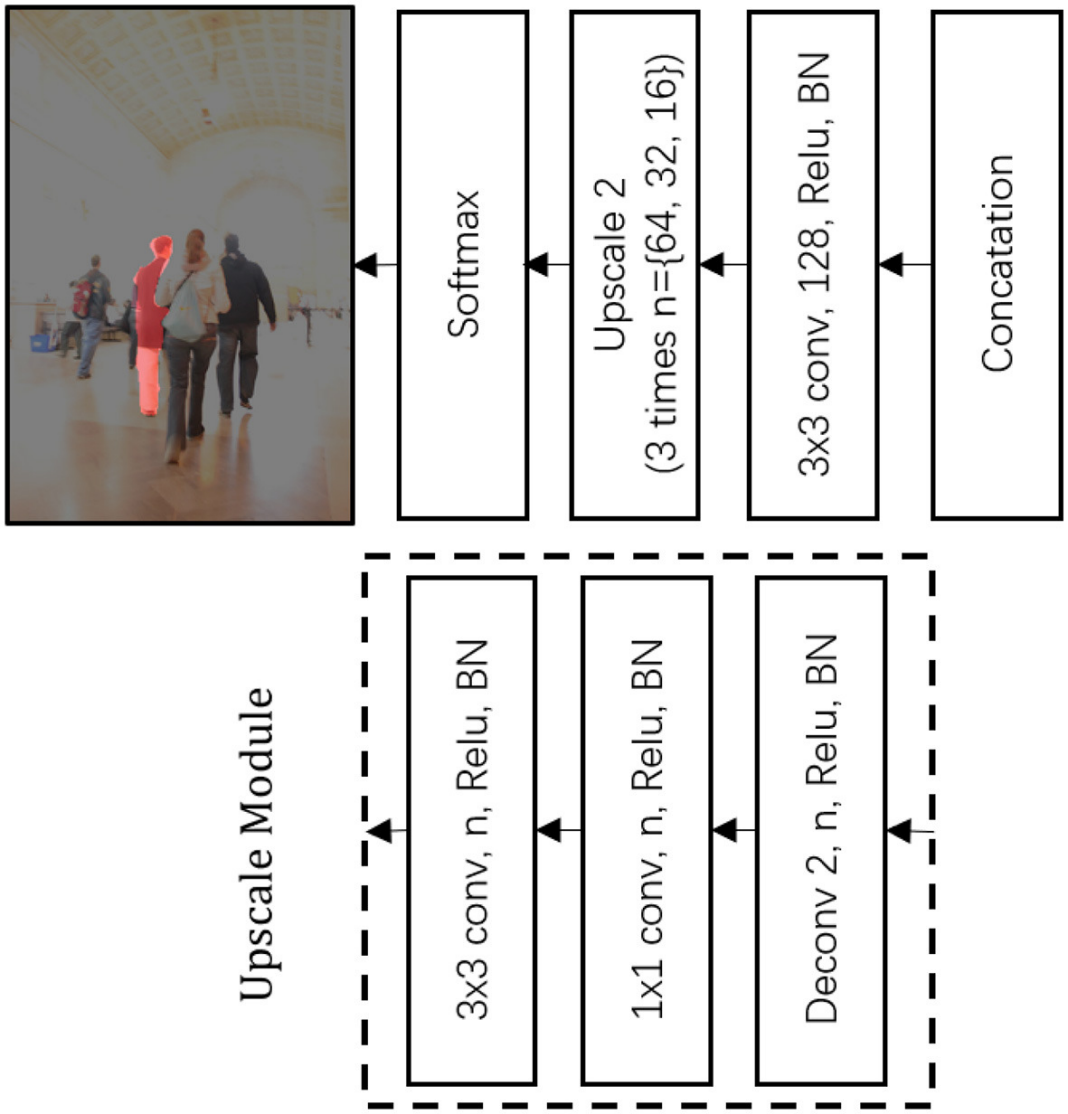
Architecture of feature merging and upscaling.

In the tracking process, our system encapsulated the distinctive model of the tracked individual into a feature map of minimal size, necessitating a mechanism to restore the original size of the segmented feature map. A schematic representation of this procedure, which includes both feature merging and upscaling, is provided in Figure 4.

At first, features derived from the discriminative correlation filter (DCF) and the discriminative geometric model (DGM), both of which are secured by a classification-lock pre-trained layer, were combined. To interpret the newly merged feature map, we used a 3 × 3 × 128 convolutional layer.

Following this, a succession of upscaling processes were executed, with each step using a deconvolution layer with an upscale size of 2, which was subsequently followed by a 3 × 3 convolution layer. This convolution layer is characterized by a progressively decreasing depth. Furthermore, it is essential to note that each convolution and deconvolution layer is accompanied by a Rectified Linear Unit (ReLU) and batch normalization (BN).

Finally, at the peak scale, the neural network generated an output via a 1 × 1 convolution layer, which was then followed by a Softmax function. This function enables the representation of the foreground target at the pixel level. Upon reaching this stage, the single shot segmentation tracker's operation comes to a conclusion, providing as its output the tracked individual encapsulated within a boundary.

### 3.4. Future motion estimation

**Figure 5:**
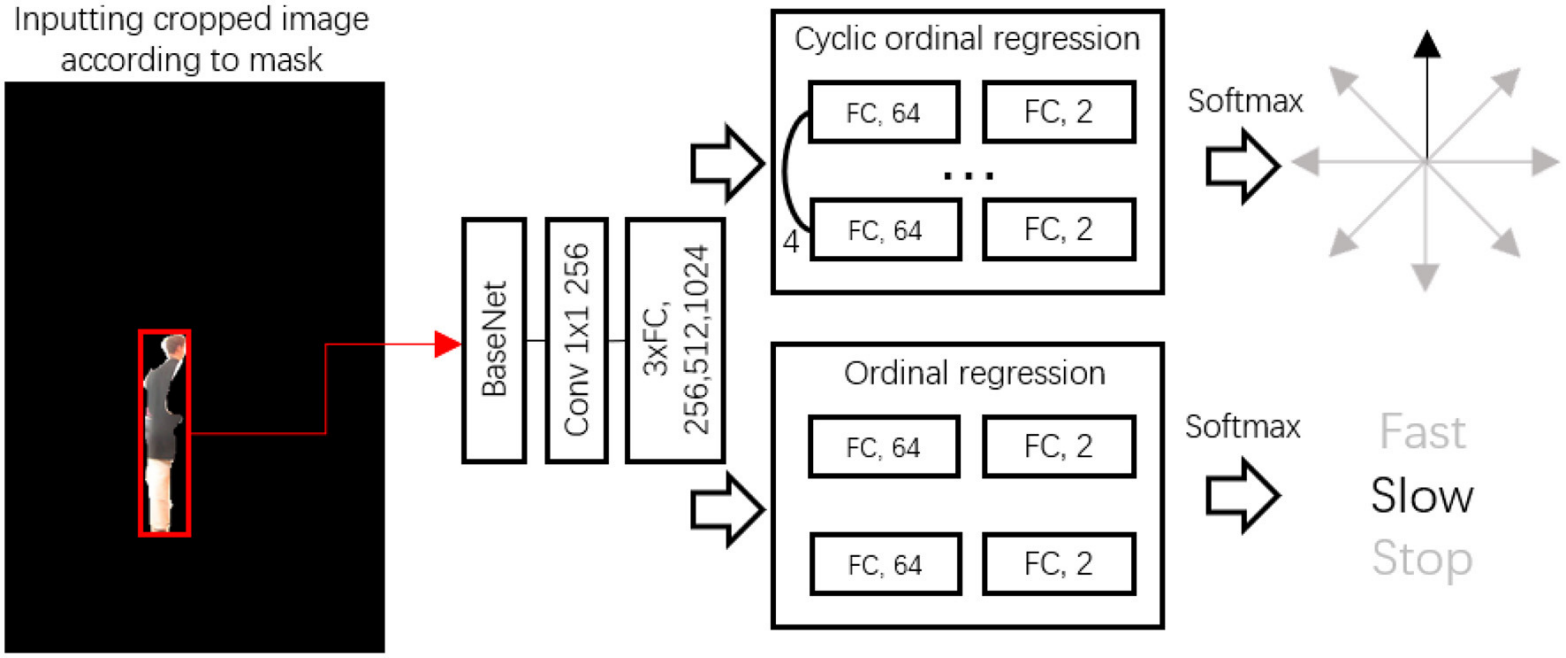
Architecture of future motion estimation.

With the boundary of the target individual now isolated, our next step involved predicting the target's future movement for the control module. In this process, we employed a future motion (FM) deep neural network (DNN) dedicated to improving future estimation. This network is unique in its ability to exceed two specific classifications: direction and action. Specifically, the FM neural network handles an image where only the individual is present in the scene. The structure of our proposed FM network is visually demonstrated in Figure 5.

Starting with the input segmentation mask, the mask region on the RGB image was utilized to crop it. This was done by extracting the top left, top height, width, and height parameters. After cropping, the area was resized to 224 by 224, ready for input into the ResNet50. From here, we employed the pooling 5 layers to fine-tune our future estimation neural network. A 1 × 1 × 256 convolutional layer served to adapt the features originating from the base network. Following this, the features were decoded using three fully connected layers.

We used two ordinal regressions to illustrate the directions and actions of the target. The future direction of the target was broken down into eight directions:

*N*(*c*_0_), *NE*(*c*_1_), *E*(*c*_2_), *SE*(*c*_3_), *S*(*c*_4_), *SW*(*c*_5_), *W*(*c*_6_), and *NW*(*c*_7_). Nonetheless, directly representing eight directions is a challenging task for the feature map. To counteract this, we applied a binary classification to determine the direction, utilizing true and false descriptions. For direction estimation, *f*_0_, *f*_1_,*f*_2_,*f*_3_, with the numbers 1 and 0 symbolizing the direction.

**Figure 6:**
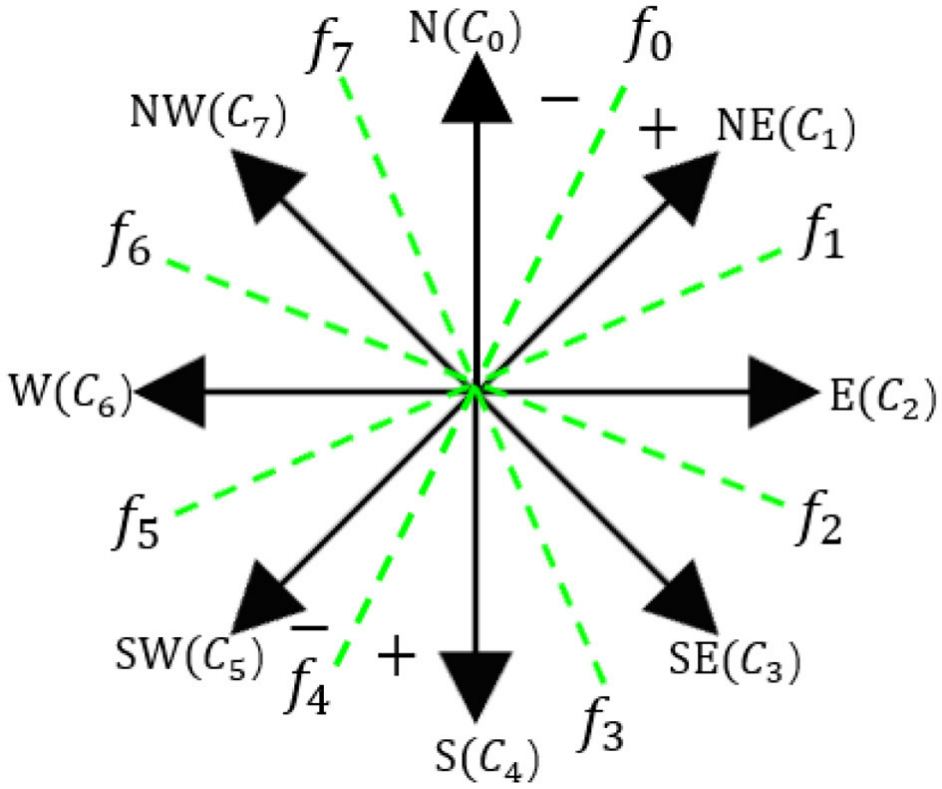
Architecture of the clockwise model.

As shown in Figure 6, directions NE, E, SE, and S are labeled 1, with all other directions labeled 0. The final direction is determined by summing the *f*values to estimate the final direction of the target, based on a cyclic order. In addition to direction, we implemented the same rule for speed estimation. This involves two binary classifiers used to estimate the motion status of the individual—whether they are moving fast, slow, or stationary. At this stage, we have successfully laid out the framework for person-following and future motion estimation.

## 4. Experiment

This section delineates the testing of our proposed framework's performance and efficiency. Initially, the tracker was evaluated utilizing the public object tracking benchmarks (Perazzi et al., 2016; Kristan et al., 2018), and compared with other contemporary trackers. Next, the efficiency of future motion estimation was assessed with and without the tracker. Lastly, the framework was implemented on a real robot platform for long-term person-following.

### 4.1. Implementation details

The models were trained on a desktop computer equipped with an AMD Ryzen 9 3900X CPU, 64 GB of memory, and a Titan RTX. The same computer was also employed for dataset evaluation. The model trained on this platform was then tested on the mobile robot. Considering the high-resolution dataset, the input size for tracking was selected as 768 × 512. For testing on the mobile robot platform, the input size was set at 384 × 256 to ensure high FPS.

#### 4.1.1. Tracker

For visual tracking, we employed ResNet 50 as the backbone network to provide pre-trained features. The YouTube-VOS dataset (Xu et al., 2018) t was used as the training segmentation sequences, where a training sample is generated by following a pair of images with corresponding masks within the last 25 frames of the video. The tracker was trained using 64 image pairs over 80 epochs with 5,000 iterations per epoch. The Adam optimizer, starting with a learning rate of 10^−3^ that decays by 0.5 every five epochs, was used. The training loss was defined by the cross-entropy between the predicted and ground-truth segmentation masks.

#### 4.1.2. Classification-lock

The classification-lock DNN was trained using image pairs from the YouTube-VOS containing human shapes, along with some of our own labeled images. A total of 2,625 images were annotated. We followed the SegNet architecture, which provides end-to-end training for the network to segment humans in the scene. The Adam optimizer was used, starting with a learning rate of 10^−4^ that decays by 0.5 every five epochs. The training loss was defined similarly to the tracker.

#### 4.1.3. Future motion estimation DNN

This network was trained on a labeled instance dataset that extends the one used for the classification-lock pre-trained DNN by adding speed and direction labels. This DNN utilized the SGDM optimizer with 0.9 momentum, a 0.1 dropout rate, and 150 epochs, starting with a learning rate of 10^−4^and a decay rate of 0.1 every 10 epochs.

### 4.2. Visual tracking evaluation

First, we tested the proposed tracker on the open source datasets VOT16/18, YouTube-VOS, and DAVIS video datasets to qualitatively evaluate its results. As the experiments are intended to be used in the person-following robot environment, we employed the evaluation merit in Zhang et al. (2019), which uses the scale and location errors to represent the quantitative results. We compared the proposed tracker with GOTURN (Gundogdu and Alatan, 2018), CFCF (Held et al., 2016), PRDIMP (Danelljan et al., 2020), and Siamese (Chen et al., 2020) in the selected video datasets. One issue remained: the compared methods and evaluation merits were in the bounding box output, but the proposed tracker was in the segmentation output. To allow it to be compared with the above methods, all segmentation masks were generated by a bounding box with min *x*, max *y*, width, and height, so as to generate a bounding box on the scene. To evaluate the results of segmentation, we compared the tracker with the state-of-the-art methods, SiamMask (Wang et al., 2019), and D3S (Lukezic et al., 2020) in the Davis video dataset with IoU, which is a common segmentation merit. It should be noted that only human-related sequences were used for the evaluation.

**Figure 7:**
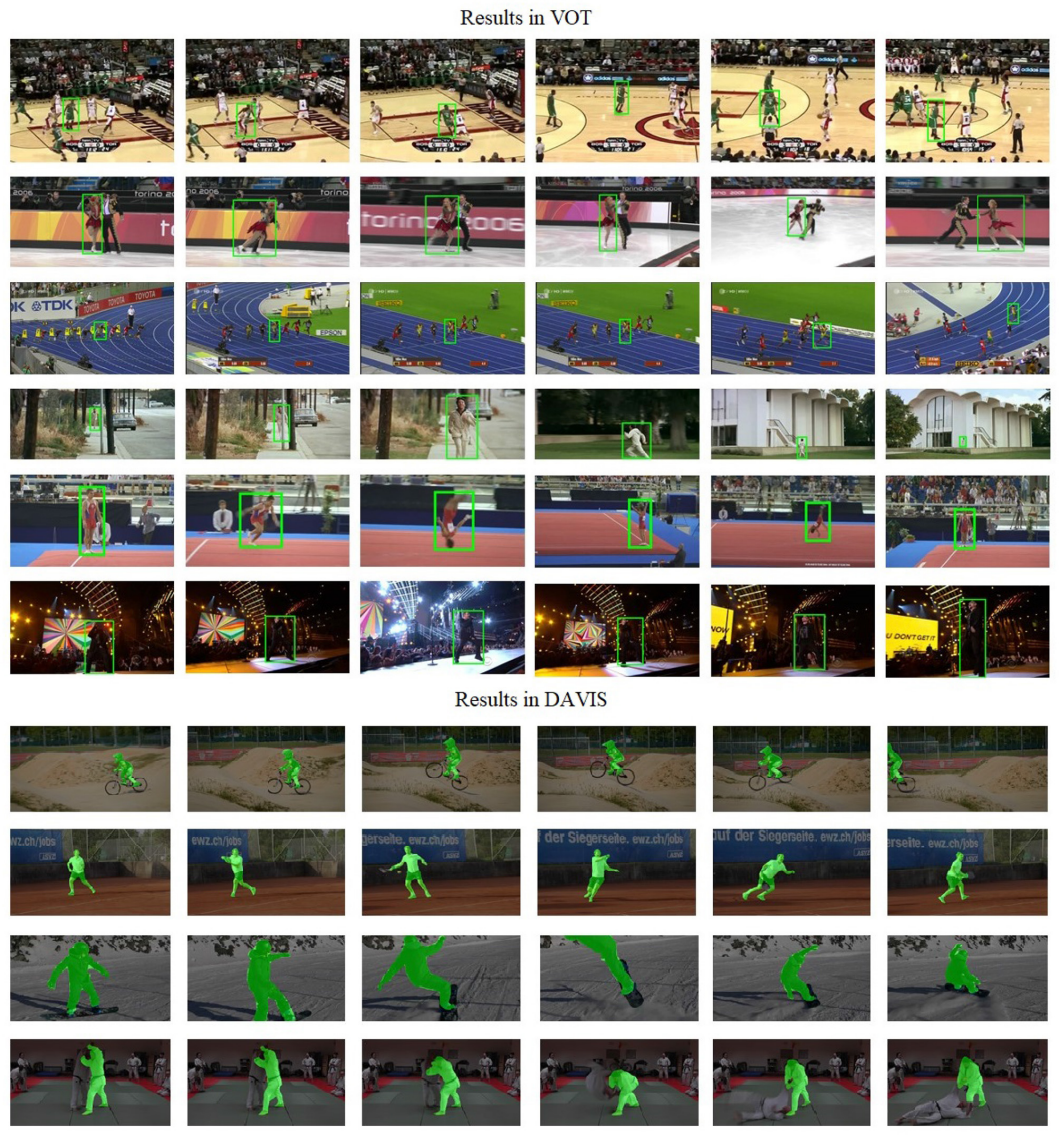
Visualization of the results in the VOT and Davis datasets.

The results of our experiments are documented in Table 1 and Figure 7. Here, MP denotes the mean average precision rate with respect to location error, and MS represents the mean average accuracy concerning scale error. NCL and WCL indicate the absence and presence of a classification-lock pre-trained layer, respectively, while FPS refers to frames per second, with “p” representing the location and scale error distances in pixels.

**Table 1:** Performance metrics for VOT 16 and 18 person objects.

**Method**	**MP (5 p)**	**MP (10 p)**	**MP (20 p)**	**MS (5 p)**	**MS (10 p)**	**MS (20 p)**	**FPS**
TLD	0.07	0.38	0.56	0.13	0.52	0.62	352.4
CMT	0.13	0.40	0.51	0.23	0.48	0.55	98.1
SPM	0.24	0.53	0.68	0.28	0.51	0.60	132.7
ATOM	0.18	0.51	0.72	0.23	0.48	0.67	29.0
GOTURN	0.15	0.52	0.77	0.20	0.43	0.63	164.0
ASRCF	0.24	0.57	0.76	0.22	0.45	0.72	27.1
CFCF	0.32	0.59	0.82	0.37	0.62	0.83	2.8
PRDIMP	0.34	0.57	0.84	0.35	0.57	0.81	43.2
SiameseRPN	0.28	0.62	0.83	0.36	0.55	0.79	37.7
Ours (NCL)	0.42	0.65	0.81	0.32	0.51	0.82	18.2
Ours	0.47	0.73	0.86	0.35	0.60	0.85	17.8

These experiments were designed in response to the operational requirements of a person-following robot, which needs to track an individual situated in the center of its field of vision and maintain a distance contingent on the person's scale. Thus, an accurate MS and MP are crucial for pinpointing the target within the scene. The results vindicate the effectiveness of the segmentation-based trackers.

Without the classification-lock pre-trained layer, our proposed method showed location errors of 0.42, 0.65, and 0.81 across 5–20 p location error distances. This significantly outperformed ROI-based trackers, especially as location error distances decreased. In stark contrast, traditional methods like TLD and CMT demonstrated extremely poor accuracy (around 0.1 in MP < 5 and MS < 5), despite achieving over 100 FPS. The segmentation tracker, with its more detailed human shape representation, allowed for more accurate target positioning.

In terms of scale error comparison, our proposed tracker without the classification-lock pre-trained layer achieved scale distance errors of 0.32, 0.51, and 0.82 from 5 to 20 p, akin to those achieved with ROI-based methods. This can be attributed to unavoidable segmentation noise during the generation of the foreground and background discriminative models, leading to similar accuracy in scale error distances. However, upon employing the classification-lock pre-trained layer, the accuracy of the proposed method improved significantly, with MP values increasing to 0.47, 0.73, and 0.86 from 5 to 20, and MS values rising to 0.35, 0.60, and 0.85, respectively, from 5 to 20. This underscores the benefits of using a human shape feature map to lock the classification for long-term tracking, providing robustness in person-following scenarios.

The visual evidence in Figure 7 also confirmed the proposed tracker's ability to overcome common person-following challenges. These include handling multiple foreground targets, drastic changes in appearance and background, changing scale, occlusions, and complicated background noise. The proposed tracker effectively navigated these challenges, mainly due to the classification-lock pre-trained layer, which provided the pre-trained human shape, minimizing appearance as an outlier factor.

As shown in Table 2, we also applied general merits from VOT 2016/18 and LaSOT to assess our method using only human items. These results further support the efficiency of the proposed method, as evidenced by our superior scores in EAO, accuracy, and robustness. In contrast, traditional methods such as TLD and CMT fail miserably, showing significantly lower scores. Our proposed method, even without the classification-lock strategy, achieved impressive results, with an EAO of 0.510, accuracy of 0.68, and robustness of 0.144. These metrics are further improved with the classification-lock strategy, obtaining an EAO of 0.538, accuracy of 0.70, and robustness of 0.140.

**Table 2:** Results of VOT 2016/2018 with only human items.

	**EAO**	**Accuracy**	**Robustness**
TLD	0.245	0.44	0.381
CMT	0.251	0.47	0.354
CMT + SegNet	0.135	0.40	0.532
SPM	0.353	0.54	0.271
ATOM	0.425	0.51	0.211
GOTURN	0.267	0.47	0.265
ASRCF	0.375	0.54	0.215
CFCF	0.354	0.56	0.225
PRDIMP	0.320	0.49	0.235
SiameseRPN	0.391	0.57	0.245
SIAMMask	0.463	0.64	0.210
D3S	0.503	0.67	0.154
SIAMRPN+ SegNet	0.328	0.45	0.288
Ours (WT)	0.510	0.68	0.144
Ours	0.538	0.70	0.140

Similar findings are reflected in the LaSOT dataset. Traditional trackers TLD and CMT continue to underperform, while DNN-based trackers with ROI output, such as SPM, ATOM, PRDIMP, and SiameseRPN, significantly outperform them. This highlights that traditional trackers and DNN-based trackers with ROI outputs struggle to handle complex human poses, while our proposed method significantly improves human tracking results, as shown in Tables 3–5.

**Table 3:** Results of LaSOT with only human items.

	**Precision**	**Norm. Prec**.	**Success (AUC)**
TLD	40.8	48.2	45.5
CMT	42.7	47.8	44.9
CMT+SegNet	35.0	41.8	42.1
SPM	52.0	56.1	51.7
ATOM	51.3	54.8	49.9
PRDIMP	56.6	60.2	53.5
SiameseRPN	57.5	62.8	56.9
SIAMMask	59.1	64.3	60.2
D3S	61.3	66.0	58.2
SIAMRPN + SegNet	47.9	52.1	48.7
Ours (WT)	62.9	68.3	61.7
Ours	65.1	70.2	62.4

**Table 4:** Performance metrics on Davis with only human items.

**Method**	**mIoU (%)**	**FPS**
SiamMask	72.3	63.0
CMT + SegNet	35.1	25.0
SiamRPN + SegNet	42.0	16.8
D3S	76.8	35.6
Ours (384 × 256)	80.2	36.4
Ours-HP (384 × 256)	78.0	108.6
Ours (768 × 512)	84.5	17.8
Ours-HP (768 × 512)	83.1	65.7

**Table 5:** Performance metrics on YouTube-VOS with only human items.

**Method**	**mIoU (%)**	**FPS**
SiamMask	67.2	63.2
CMT + SegNet	40.5	23.5
SIAMRPN + SegNet	43.5	17.5
D3S	72.5	35.7
Ours (384 × 256)	76.3	36.2
Ours-HP (384 × 256)	74.5	112.3
Ours (768 × 512)	81.0	17.9
Ours-HP (768 × 512)	79.9	68.6

To further delve into the merits of tracking-by-segmentation methods, we examined the Davis and YouTube-VOS datasets. As shown on Table 4 and 5, here, our tracker attained 84.5 and 81.0% mIoU in Davis and YouTube-VOS respectively, vastly outperforming D3S and SiamMask. Although the FPS of our method is lower, it is mainly due to the doubling of the input resolution. With the same input size (384 × 256), our method obtained a mIoU of 80.2%/76.3% and an FPS of 36.4/36.2, surpassing the state-of-the-art methods. Employing TensorRTX with half-precision settings, our method attained real-time functionality on robots while suffering only a 2% reduction in accuracy.

When considering the unique strengths and applicability of our “tracking-by-segmentation” approach, it is paramount to understand that this technique delivers superior performance in capturing precise representations of a target's appearance, an aspect that is crucial in person-following tasks.

Unlike ROI-based methods, which often struggle to accurately capture the target's distinctiveness within the scene, our approach adeptly addresses this challenge. This is achieved by taking advantage of segmentation rather than relying solely on the basic bounding boxes used by most current methods.

The advantage is not merely a technical nuance but results in a substantial improvement in the practical performance of person-following tasks. The segmentation method provides a more refined understanding of the target's shape and characteristics, enabling the system to maintain effective tracking even when dealing with significant variations in appearance, scale, and pose.

Furthermore, the unique strength of our method lies in its adaptability and robustness to complex environments, which pose a significant challenge to traditional ROI-based methods. Our approach ensures consistent performance in scenarios like handling occlusions, changes in lighting, or background noise, resulting in improved tracking accuracy and overall system stability.

As our comprehensive set of experiments on various challenging datasets suggests, our approach outperforms its counterparts. The “tracking-by-segmentation” technique represents a paradigm shift in dynamic tracking for person-following robots. This comparison aims to illustrate the substantive contribution of our method compared to existing state-of-the-art solutions.

Finally, we compared the performance of our method using different base network architectures, such as ResNet 50, ResNet 18, MobileNet V3, and Inception V4, on the Davis dataset. The method using ResNet 50 achieved the most balanced performance and was therefore selected as the base network. Overall, the proposed framework demonstrates robustness in overcoming common challenges regarding person-following. Nevertheless, it is essential to conduct real-world robot experiments to further evaluate the performance of the proposed method, as shown in Table 6.

**Table 6:** BaseNet selection in DAVIS.

**Method**	**mIoU (%)**	**FPS**
ResNet 18	79.2	30.4
Inception V4	84.8	6.9
MobileNet V3	72.6	36.0
ResNet 50	84.5	17.8

Another concern is the basenet selection. We compared the proposed method in ResNet 50, ResNet 18, MobileNet V3, and Inception V4 in the Davis dataset. The results are shown in Table 3. The method using ResNet 18 obtained 79.2% mIoU and can operate at 30.4 FPS. Although the FPS of ResNet 18 is higher than that of ResNet 50, the accuracy is 5% lower. The Inception V4 obtained 84.8% mIoU and 6.9 FPS. Although the accuracy is higher than the proposed basenet, the FPS is too slow to operate on the mobile platform. Mobilenet V3 obtained 72.6% mIoU and 36.0 FPS. Although the FPS is much faster than the proposed method, its accuracy is the lowest among the compared methods. In the evaluation, ResNet 50 obtained the most balanced performance, so we selected ResNet 50 as the basenet.

### 4.3. Evaluation of a real-world robot platform

**Figure 8:**
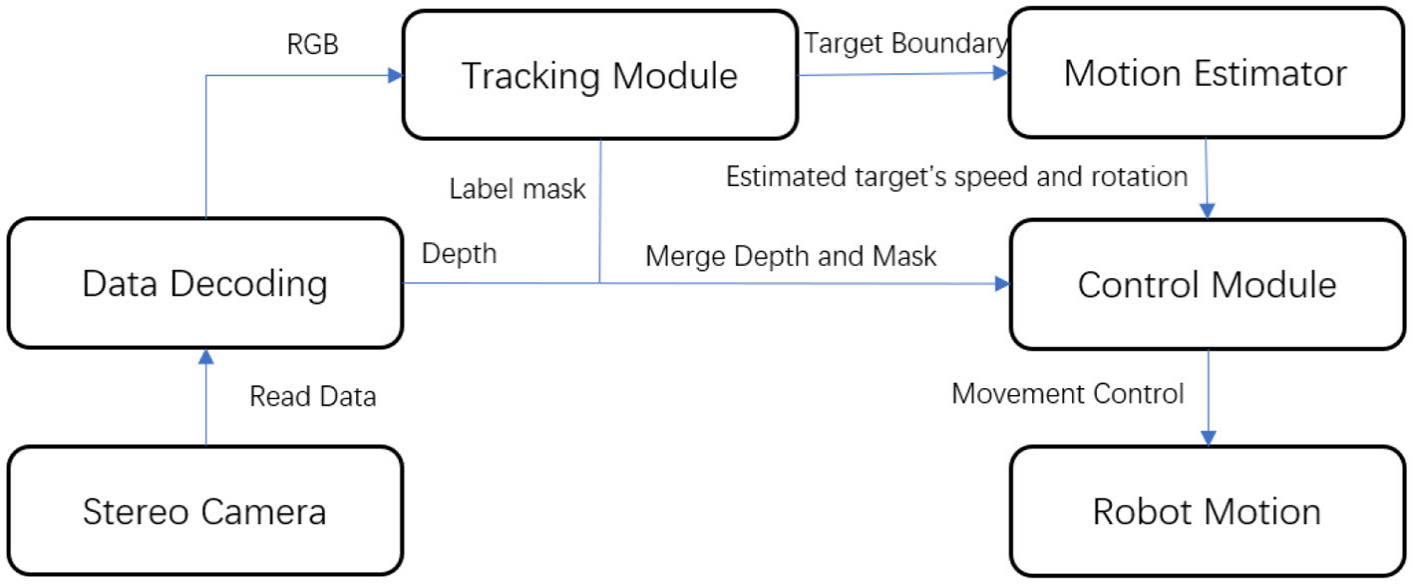
Framework of the robot platform.

We constructed and employed a mobile robot platform for real-world robot experimentation. We evaluated the future motion estimation experiment in conjunction with real-world person-following tasks on this platform. The robot was built on an underpinning and outfitted with eight independent suspensions and steering wheels for all-terrain movement. The robot's body had a stable lifting capacity, ranging from 100 to 150 cm, to adjust the camera's view of the human subject. The robot's visual system utilized a ZED camera to gather both RGB and disparity information from the scene. A laptop computer with an I7-9850H CPU, 32 GB of DDR-4 memory, and a mobile RTX 2080 served as the robot's control unit. The overall person-following framework is illustrated in Figure 8.

The robot subscribes to the nodes from the stereo camera, encoding them into RGB and depth signals. These RGB signals are then input into the tracking module to track the target. The module provides two outputs: motion estimation and target boundary masks. The extracted target mask is merged with the depth signal, and the center of mass is chosen as the target's position. The mean value of the pixels of the mask region on the depth map is calculated to determine the distance to the target. This position and depth information is then fed to the control module, together with the speed and direction data from the motion estimation.

In our system, we utilized a classic Proportional-Integral-Derivative (PID) controller, due to its low computational overhead, ease of implementation, and reliability in real-time control on mobile platforms. While we acknowledge the potential of methodologies like Shi et al. (2023a) and Shi et al. (2023b), their real-time applications on limited-resource platforms remain challenging. We applied the penalty from future motion estimation (speed and direction) to adjust the robot's rotation and speed. Initialization was performed remotely, using a mobile phone connected to the laptop computer to provide an initial bounding box. The system did not incorporate a re-detection module as the tracking-by-segmentation DNNs should, in principle, not lose tracking. The robot was set to maintain a fixed distance of 1.5 m from the target person, and it considered the task completed when the distance was reduced to < 0.5 m and the person was facing the robot.

Subsequently, we assessed the future motion estimation feature in the robot system. We used the precision rate to evaluate whether the speed and pose were correct duringwhen it comes to real-world robot deployment tracking based on a real person-following video with 1438 frames. We compared our method with several existing ones, such as the ones by HOG (Dalal and Triggs, 2005), ACF (Dollár et al., 2014), Gao et al. (2018), and Kim et al. (2019). Our method demonstrated considerable accuracy and performance improvements over traditional methods, as shown in Table 7.

**Table 7:** Evaluation of future motion estimation.

**Method**	**Speed**	**Direction**	**FPS**
HOG	0.32	0.42	62
ACF	0.42	0.47	58
Kim et al.	0.60	0.61	51
Gao et al.	0.61	0.60	50
Ours	0.85	0.86	243

We attributed these improvements to the advantages our method has over traditional ones with the future motion estimation dataset (Kim et al., 2019). The comparative data is detailed in Table 7, which demonstrates the varying levels of accuracy between different methods. Traditional feature-based methodologies typically employ a combination of a sliding window approach and a classifier to determine speed and direction. However, even though they can function at over 60 frames per second (FPS) exclusively using the CPU, the accuracies they yield in both speed and direction estimation are considerably lacking. Notably, Histograms of Oriented Gradients (HOG) and Aggregate Channel Features (ACF) methods fall into this category.

On the other hand, convolutional neural network (CNN)-based techniques, such as those proposed by Gao et al. and Kim et al. showcase improved accuracies surpassing 0.6 in both categories, and they achieve approximately 50 FPS when employing the GPU. Nevertheless, these methods present their own set of challenges when it comes to real-world robot deployment. Two major obstacles can be identified: first, they consume considerable GPU resources, which can adversely affect the functioning of other operational components within the robot's operating system. Second, the accuracy they provide is not consistently reliable, which in turn complicates the decision-making process for robot control.

Another fundamental shortcoming lies in target localization. These methods necessitate the identification of potential target positions and regions before classifying speed and direction, which substantially delays response times. In contrast to these methodologies, our approach relies on continuous tracking derived from tracking-by-segmentation results. This strategy significantly reduces computation time and guarantees a high level of speed and direction estimation as only a few proposals are required for detection.

Our method achieves superior accuracy rates of 0.85 for both speed and direction, operating at a higher frame rate of over 240 FPS. This evaluation reinforces the conclusion that our approach is the most suitable for person-following robots, given their superior performance and efficiency.

**Figure 9:**
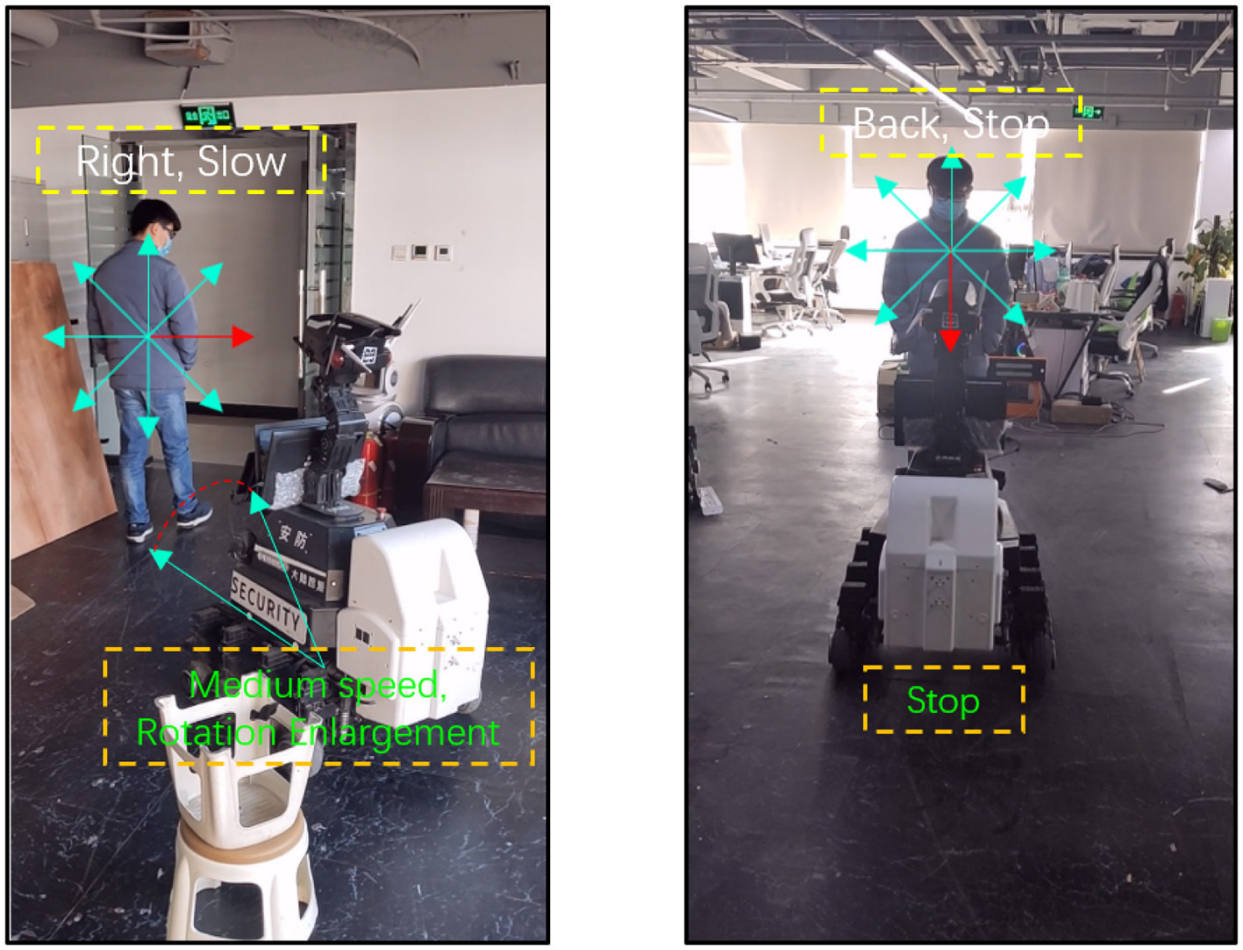
Rotation and stop sample.

In the att ached demo videos, we have performed a comparative evaluation of the use of traditional trackers such as CMT, ROI-based trackers based on DNNs (SiamRPN), general tracking-by-segmentation trackers (D3S), and our method on a real-world robot movement. The CMT failed due to loss of tracking caused by sensitive background. The SiamRPN also failed due to the limitations of ROI in accurately describing human appearance. D3S also encountered issues due to low FPS and false positive appearance boundaries during tracking. In contrast, our method with the classification-lock strategy maintained the focus on the human target and provided a robust tracking result to the controller. Demo is shown on Figure 9.

Our system's effective handling of the robot's rotation is largely due to the incorporation of future motion estimation. When the robot is tracking the target, it is not uncommon for the target to make abrupt changes in direction. Without future motion estimation, these sudden changes can cause the robot to overcompensate in its rotation, often leading to inefficient movement or even losing sight of the target altogether.

**Figure 10:**
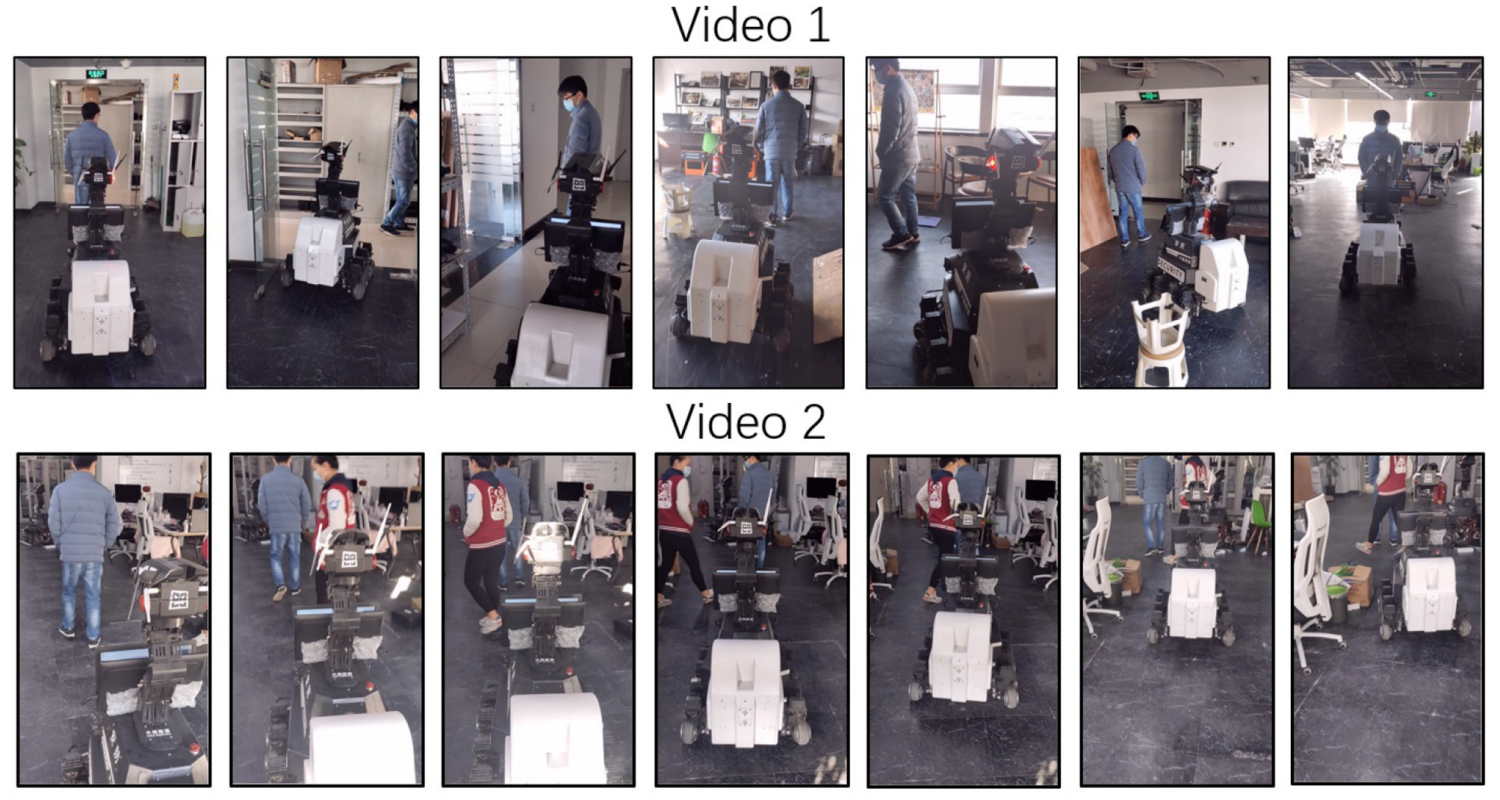
Person-following video.

However, with the future motion estimation implemented in our model, the robot is able to predict these sudden turns. This allows the robot to adjust its rotation more smoothly, resulting in less drastic rotations and closer adherence to the target's path. The importance of this feature is highlighted in Figure 10, which shows a marked difference in the degree of rotation when future motion estimation is employed compared to when it is not.

Furthermore, our future motion estimation DNN provides an accurate motion estimation indicator for the control module. This facilitates more optimal motion tracking, as it equips the robot with the ability to accurately anticipate the target's motion and plan its route accordingly.

Moreover, the tracking module consistently tracks the target despite changes in appearance, rotation, and in scale. This is demonstrated in the accompanying videos, where the robot is shown successfully following the target even as it moves, rotates, changes shape, and experiences varying lighting conditions in a complex environment. This combination of the tracking module and future motion estimation ensures robust, efficient tracking in real-world conditions.

In the second video, we can observe that the robot robustly tracks the person even when the individual frequently overlaps with another person. This is because the tracking-by-segmentation-based tracker allows for pixel-level tracking, which is not affected by overlapping, and still maintains focus on the correct target to ensure robust following.

Through the two videos, we demonstrated that the proposed method robustly tracks the target person in a complex indoor environment, overcoming various challenging influences. The results support our assertion that the classification-lock strategy combined with tracking-by-segmentation keeps the tracker's focus on the human, ensuring reliable tracking results for the controller. This makes our method particularly well-suited for person-following tasks on real-world robot platforms.

## 5. Demo

The detailed demo can be found at:

https://1drv.ms/u/s!AgR9F-D39FR-zBROVPTb2D-AzLgO?e=AddEMi.

## 6. Conclusions and discussion

Throughout this manuscript, we have detailed an innovative approach to person-following robots that interweaves aspects of visual tracking, object segmentation, pixel-level classification-lock strategy, and future motion prediction to achieve remarkable performance improvements.

Our proposed framework significantly outperforms existing solutions on the VOT, LaSot, YouTube-VOS, and Davis datasets, especially in the context of human tracking. The novel introduction of the classification-lock strategy, reinforced by a pre-trained layer within the architecture, has resulted in a significant improvement in tracking accuracy. This is further underscored by the tracker's precision in handling the complex nature of person-tracking.

The future motion estimation aspect of our deep neural network (DNN) has provided convincing evidence of its capability to offer precise estimations during tracking. This foresight, particularly in anticipating rotations, has significantly improved the robot's ability to adapt and respond to changes in both the environment and the target's behavior.

We also conducted real-world robot testing, which showcased the tracker's impressive ability to maintain continuous tracking, even in the face of common challenges like occlusion. This practical validation highlights the robustness and applicability of our proposed solution in complex environments.

However, we acknowledge that an exploration of potential challenges and subtleties in more dynamic environments, such as industrial or outdoor settings, remains to be done. Future research may focus on enhancing the robustness of our tracking system under different conditions and striking an optimal balance between prediction accuracy and computational efficiency.

In conclusion, our research offers a groundbreaking approach that fuses tracking-by-segmentation with future motion estimation to dramatically increase the robustness and efficiency of person-following tasks in indoor service robots. This pioneering work, combined with our reflections on the methodology in comparison with existing approaches and suggestions for future research directions, contributes to the body of knowledge in this field and could set a new standard for future studies.

## Data availability statement

The original contributions presented in the study are included in the article/supplementary material, further inquiries can be directed to the corresponding author.

## Author contributions

SJ: Investigation, Methodology, Project administration, Resources, Software, Supervision, Validation, Visualization, Writing—original draft, Writing—review and editing. RC: Conceptualization, Data curation, Formal analysis, Writing—original draft. RW: Methodology, Writing—original draft. ZF: Data curation, Investigation, Writing—original draft. ZH: Investigation, Supervision, Writing—original draft, Writing—review and editing. GF: Supervision, Writing—original draft, Writing—review and editing.
